# Congenital heart disease in school-aged children: Cognition, education, and participation in leisure activities

**DOI:** 10.1038/s41390-021-01853-4

**Published:** 2021-12-01

**Authors:** Rebecca Spillmann, Susanne Polentarutti, Melanie Ehrler, Oliver Kretschmar, Flavia M. Wehrle, Beatrice Latal

**Affiliations:** 1 Child Development Center, University Children’s Hospital Zurich, Zurich, Switzerland; 2 Department of Pediatric Cardiology, Pediatric Heart Center, University Children’s Hospital Zurich, Zurich, Switzerland; 3 Children’s Research Center, University Children’s Hospital Zurich, Zurich, Switzerland; 4 University of Zurich, Zurich, Switzerland; 5 Department of Neonatology and Intensive Care, University Children’s Hospital Zurich, Zurich, Switzerland

## Abstract

**Background:**

Children with congenital heart disease (CHD) are at risk for neurodevelopmental deficits. This study aimed to investigate the impact of cognitive deficits on educational outcome and participation in leisure activities.

**Methods:**

A prospective cohort of 134 children with CHD who underwent cardiopulmonary bypass surgery (CPB) was examined at 10 years of age. IQ was assessed with the WISC-IV and executive functions with the BRIEF (parent- and teacher-report). Parents reported on type and level of education and educational support, and leisure activity participation. Ordinal regression analyses assessed the association between cognitive deficits and educational outcome and participation.

**Results:**

Total IQ (*P* = 0.023), working memory (*P* < 0.001), processing speed (*P* = 0.008*)*, and teacher-reported metacognition (*P* = 0.022) were lower than norms. Regular school was attended by 82.4% of children with CHD compared to 97% of the general Swiss population (*P* < 0.001). Seventy-five percent of children participated in leisure activities. Lower total IQ and teacher-rated global executive functions were associated with more educational support and lower IQ was associated with less participation.

**Conclusion:**

As school-aged children with CHD experience cognitive deficits, follow-up is required to provide optimal support with regard to educational outcome and participation in leisure activities.

**Impact:**

- Contemporary cohorts of children with congenital heart disease undergoing cardiopulmonary bypass surgery remain at increased risk for cognitive deficits.
- Cognitive deficits affect educational outcome and leisure activities.
- These findings underline the importance of early detection of cognitive deficits and recommend support with respect to cognitive functioning.

## Introduction

Congenital heart disease (CHD) is the most frequent congenital malformation, affecting 8 in every 1000 live births.^1^ Children with severe CHD requiring cardiopulmonary bypass surgery (CPB) are at risk for a variety of neurodevelopmental sequelae: On group level, IQ scores are frequently reported to be lower than, but within the average range of the population norm or compared to typically-developing control groups.^2–7^ Alongside lower IQ, deficits in executive functions—higher-order cognitive skills required for behavioral control and change of habits^8^—are also frequently reported in children and adolescents with CHD.^9–12^

In typically-developing children, higher IQ and better executive functions are linked to better educational outcome.^13–16^ While a number of studies have reported poorer academic performance and higher rates of special education services and grade retention in children with CHD,^17–19^ studies linking IQ and executive functions with education are limited in this population and results are mixed. For example, one study found IQ and executive functions to predict school competence in children with CHD^5^ while another reported no evidence that executive dysfunctions are linked to receiving special educational services.^9^ Interestingly, one study reported better cognition to be related to higher levels of physical and social activity participation in children and adolescents with CHD.^20^ This further illustrates the potential impact of neurodevelopmental sequelae on these patients’ everyday lives.

While operative procedures and perioperative care continue to improve, it is unclear how this impacts the neurodevelopmental outcomes of children with CHD.^21^ Thus, the long-term evaluation of contemporary cohorts is essential and helps to understand how potential impairments may impact everyday life in these patients. Consequently, the aims of this study were (i) to describe the IQ and executive function profile, the type and level of education and educational support, and the participation in leisure activities in a contemporary cohort of 10-year-old children with CHD who underwent CPB surgery between 2004 and 2009. We hypothesize IQ, executive functions, educational outcome, and participation in leisure activities to be lower compared to population norms. Further, we aimed (ii) to investigate whether cognitive deficits are associated with education and participation. We hypothesize lower IQ and poorer executive functions to be linked to higher levels of educational support and lower levels of participation in leisure activities.

## Methods

### Participants and study procedure

The Research and Child Health Outcome (REACHOUT) study prospectively included and longitudinally followed 300 children with CHD who underwent CPB surgery between 2004 and 2009 at the University Children’s Hospital Zurich, Switzerland. Inclusion criteria were CPB surgery after enrollment and first surgery before the age of 6 years. Neurodevelopmental assessments took place prior to the surgery and at 1, 4, 6, and 10 years of age. Between the enrollment and the 10-year follow-up, 27 children died: Four children died within the first 30 days and nine within the first year in the hospital after surgery due to cardiac failure (*n* = 9) or cerebral hemorrhage, septic shock, intraoperative bleeding, or restricted ventilation (*n* = 1). Five children died because of cardiac failure not linked to the surgery and two because of severe respiratory infections within the first year. Five patients died within the first 4 years of life because of cardiac failure (*n* = 4) or pulmonary hypertensive crisis (*n* = 1). Two children died at an older age (7 and 9 years of age) due to cardiac failure not linked to surgeries.

**Fig. 1:**
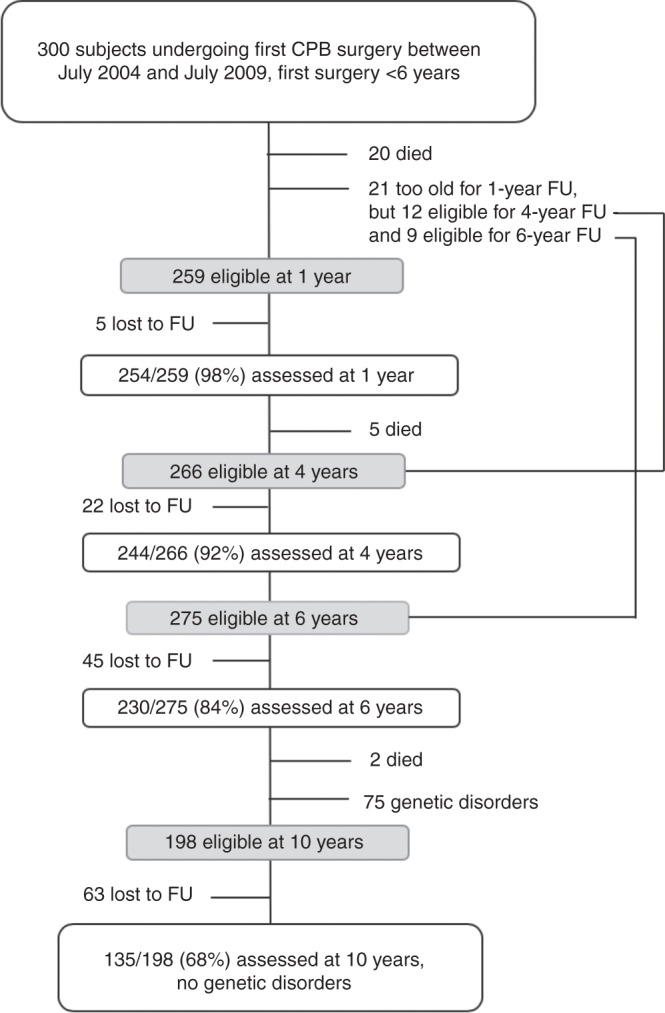
Flowsheet of the population assessed at 10 years of age. CPB Cardiopulmunary bypass surgery, FU follow-up, y year.

At the 10-year follow-up assessment, children with a genetic or syndromal disorder were excluded from the prospective study cohort (*n* = 75 children). This resulted in 198 children eligible for the 10-year follow-up. Sixty-three children were lost to follow-up. Thus, 135 children were assessed at 10 years of age (for a flow chart see Fig. 1). Children who participated had more severe forms of CHD than children lost to follow-up (cyanotic: 68.1% vs. 42.9%, *P* < 0.001; univentricular: 21.5% vs. 4.8%, *P* = 0.003). At 6 years of age, mean IQ was higher in the 135 participating children than in the non-participating children without a genetic or syndromal disorder (95.6 ± 12.7 versus 85.3 ± 16.2, respectively, *P* = 0.001). No difference in socioeconomic status (SES) was found between these two groups (median = 8 in both groups; *U* = 2957.5; *P* = 0.097).

Over the course of ~3 h, each child underwent a comprehensive neurodevelopmental assessment by a pediatric neuropsychologist who was aware of the cardiac history of the patient. One child was excluded from further analyses because the test applied to assess cognition deviated from the REACHOUT study protocol. Therefore, the study presented here reports on 134 children at the age of 10 years. This study was approved by the ethics committee of the Canton of Zurich, Switzerland, and written informed consent was given by the parents.

### Assessment of IQ, executive functions, education, and participation

The German version of the Wechsler Intelligence Scale for children—Fourth Edition (WISC-IV) was used to evaluate total IQ and the indices verbal comprehension, perceptual reasoning, working memory, and processing speed.^22^ IQ below 85 (norm 15.9%) was considered abnormal. The Behavior Rating Inventory of Executive Functions (BRIEF)^23^ was used to assess executive functions in everyday situations. Scores are expressed as age- and sex-adjusted *T*-values, with higher scores representing worse executive functions.^23^ Scores of 65 or higher (norm 6.7%) are considered clinically significant executive dysfunctions. For the current analyses, the global executive functions score and the indices metacognition and behavioral regulation were used. In total, 118 (88.1%) parents and 114 (85.1%) teachers completed the BRIEF.

Parents reported on the type and level of education and educational support their child had received in the past or was currently receiving (*n* = 125; 93.2%). In Switzerland, the vast majority of children is enrolled in the free-of-charge public educational system: Two years of kindergarten start at four years of age (in some cantons, only one year is compulsory, and in some cantons, kindergarten and the first one or two years of primary school are summarized in a three- or four-year basic level (Grundstufe)). After kindergarten, six years of primary school follow. Children with special educational needs are integrated into regular schools whenever possible. Specialized staff is assigned to the child for some hours per week. If integrative school is not possible, children are enrolled in designated schools for children with special educational needs. Academic tutoring in addition to regular schooling is partly offered by schools but frequently organized and financed individually by parents. After primary school, children are assigned to different levels of secondary school: While the higher levels enable enrolment at a university, the lower levels enable an apprenticeship. For the current study, a score was developed to reflect the overall type and level of education of each child on a 4-point scale: “Attendance of a school for children with special needs”, “integration with educational needs into a regular school”, “attendance of regular school with additional educational support” or “attendance of regular school without additional support”. Schools for children with special needs include special educational schools in general, speech therapy schools, and schools with a reduced number of children (Kleinklasse). For this study, private schools were also grouped into this category. Additional educational support includes an additional year of schooling (e.g., three years of kindergarten, grade retention) and additional tutoring. Similar factors have previously been considered in other studies evaluating type and level of education and educational support in very preterm and full-term children and adolescents.^24,25^

Parents also reported on participation in leisure activities (*n* = 125; 93.2%). In Switzerland, leisure activities are generally organized independent of schools, e.g., as private clubs. While some leisure activities are available only in larger cities, many are widely available, both in urban and rural areas (e.g., soccer clubs, scouts, guitar classes). Participation fees vary considerably between different leisure activities and are covered by the parents. In the current study, parents reported whether their child participated in any sports or another type of leisure activity (e.g., scouts) or played a musical instrument at least every other week. Participation across the different activities was summed and categorized as participation in no, one, two, or three or more activities for further analyses. Missing questionnaires were mostly due to parental language difficulties.

SES was assessed at birth and measured by maternal education and paternal occupation on a scale ranging from 2 to 12, with 12 indicating the highest SES.^26^

### Statistical analyses

Descriptive statistics of demographic, medical, and pre-, intra- and postoperative characteristics, and IQ and executive functions include numbers and percentages of the total, median, and range for categorical variables and mean (*M*) and standard deviation (SD) for continuous variables. Means were compared against normative values (IQ: *M* = 100, SD ± 15 and executive functions *M* = 50, SD ± 10) as provided by the respective manuals^22,23^ using Welch modified two-sample *t*-test, allowing for unequal variance. Chi-square test was used to compare type and level of education to representative data from the Swiss Federal Statistical Office (2017/18), which reports on the education of 511,415 Swiss children from 1^st^ to 6^th^ grade, corresponding approximately to ages 6–12 years.^27^ No normative data for participation in leisure activity was available, thus, no statistical comparison to Swiss data was possible. However, participation in sports was compared descriptively to the data provided by the Sport Switzerland study (2008/2014). This study reported on weekly sports activity of three or more hours in ~1530 representative Swiss children between the age of 10 and 14 years.^28,29^

Multivariate linear regression models were used to investigate the impact of medical risk factors and SES on IQ and executive functions. The selection process of the respective medical risk factors has been described in detail before.^30^ In short, risk factors reported in CHD patients were gleaned from a literature search. Then, these risk factors were explored in the current data set and the number was reduced according to clinical considerations to avoid collinearity. This resulted in the following medical and neonatal risk factors: Gestational age (GA, in weeks), birth weight (*z*-score, corrected for GA), univentricular CHD (yes/no), mean preoperative saturation (in %), age at first CPB (in months), lowest perioperative temperature (in °C), extracorporeal circulation during the first CPB surgery (in minutes), and length of hospitalization (in days). *R*^2^, standardized regression coefficients as *β*, respective CI-95, and *P*-values were reported for the multivariate linear regression models.

Ordinal regression was used to investigate the impact of IQ, executive functions, and SES on the type and level of education and on participation in leisure activities. Log odds as estimates (*B*), the respective 95% confidence interval (CI-95), and *P*-values are reported. *P*-values ≤ 0.05 were considered significant. Statistical analyses were performed using *R* (version 3.5.1)^31^ and SPSS 24.

## Results

### Neurodevelopmental outcome

The mean age of the study cohort of 134 children with CHD was 10.2 ± 0.25 years at the time of the assessment. Demographic, medical, and pre-, intra-, and postoperative data are presented in Table 1.

**Table 1:** Demographic, medical, and pre-, intra-, and postoperative characteristics of 134 children with congenital heart disease.

Demographic and medical variables^a^	
Age at assessment (in years), *M* (SD),	10.2 (0.27)
Male, *N* (%)	80 (59.7)
Socioeconomic status, median (range)	8 (3–12)
Caucasian race, *N* (%)	126 (94.0)
Prenatal diagnosis, *N* (%)	28 (20.9)
Gestational age (in weeks), *M* (SD)	39.01 (2.13)
Weight, *M* (SD)	
Birth weight (in grams)	3187.44 (638.91)
Birth weight (*z*-scores)	−0.39 (1.14)
Weight at 6 years of age (*z*-scores)	−0.29 (1.08)
Weight at assessment (*z*-scores)	−0.22 (1.14)
Head circumference, *M* (SD)	
Head circumference at birth (in cm)	34.18 (1.68)
Head circumference at birth (*z*-scores)	−0.53 (1.10)
Head circumference at 6 years of age (*z*-scores)	−0.66 (1.21)
Head circumference at assessment (*z*-scores)	−0.52 (1.29)
Length, *M* (SD)	
Length at birth (in cm)	48.91 (2.86)
Length at birth (*z*-scores)	−1.00 (1.04)
Length at 6 years of age (*z*-scores)	−0.21 (.95)
Length at assessment (*z*-scores)	−0.10 (1.03)
5-min Apgar score, median (range)	9 (1–10)
Cardiac class, *N* (%)^c^	
1. 2 Ventricles/no aortic arch obstruction	94 (70.1)
2. 2 Ventricles/aortic arch obstruction	12 (9.0)
3. Single ventricle/no aortic arch obstruction	12 (9.0)
4. Single ventricle/aortic arch obstruction	16 (11.9)
Cyanotic heart defect, *N* (%)	91 (67.9)
Univentricular heart defect, *N* (%)	28 (20.9)

Preoperative^a,b^	
Age at first surgery, months, median (range)	1.45 (0–36)
Surgery during neonatal period (≤30 days), *N* (%)	63 (46.6)
Preoperative cyanosis, *N* (%)	48 (35.8)
Preoperative intubation, *N* (%)	21 (15.7)
Preoperative hematocrit (%), *M* (SD)	41.14 (6.64)
Preoperative saturation (%), *M* (SD)	86.35 (10.25)
RACHS score, median (range)	3 (1–6)

Intraoperative^a,b^	
Lowest temperature during first surgery, (°C), *M* (SD)	29.07 (4.16)
Lowest saturation during first surgery (%), *M* (SD)	23.69 (5.57)
Hypothermia (<28 °C), *N* (%)	26 (19.4)
ECC time during first surgery, *M* (SD)	165.60 (72.31)
Aortic cross-clamping time during first surgery, (in min), *M* (SD)	84.84 (45.33)

Postoperative^a,b^	
Clinical seizures after first surgery, *N* (%)	1 (0.8)
ECMO postoperative, *N* (%)	2 (1.5)
Cardiocirculatory resuscitation postoperative, *N* (%)	10 (7.5)
Length of ICU-stay after first surgery, (in days), *M* (SD)	11.85 (21.33)
Total number of surgeries, median (range)	1 (1–4)
Total number of catheter interventions, median (range)	1 (0–11)

*ECC* extracorporeal circulation, *ECMO* extracorporeal membrane oxygenation, *ICU* intensive-care-unit, *RACHS* risk adjustment for congenital heart surgery.

^a^Missing data: socioeconomic status: *n* = 3; gestational age: *n* = 2; birth weight: *n* = 1; head circumference at birth: *n* = 21; head circumference at 6 years of age: *n* = 6; head circumference at 10 years of age: *n* = 7; length at birth: *n* = 4; length at 6 years of age: *n* = 4; length at assessment: *n* = 1; 5-min Apgar score: *n* = 12; preoperative hematocrit: *n* = 2; preoperative saturation: *n* = 8; lowest saturation during first surgery: *n* = 1; aortic cross-clamping time during first surgery: *n* = 11; clinical seizures after first surgery: *n* = 2; ECMO postoperative: *n* = 1.

^b^Variables referring to the first cardiopulmonary bypass surgery.

^c^Cardiac class according to Clancy et al.^47^.

#### IQ and executive functions

**Fig. 2:**
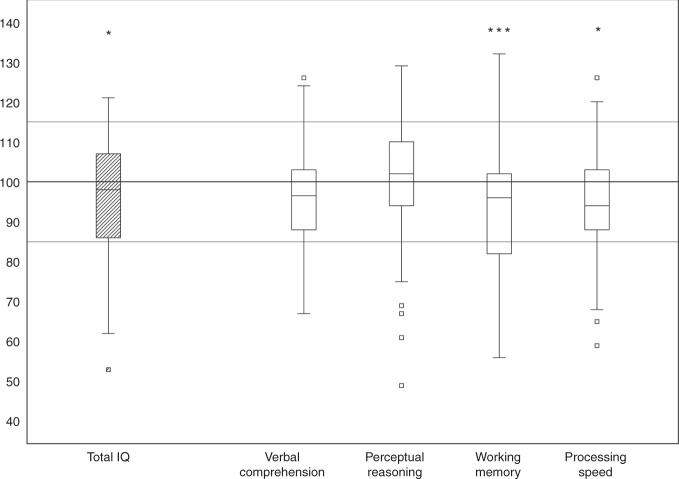
Cognitive outcome measured with WISC-IV, German version. Boxplots for total IQ and the 4 indices are presented. The line within the box is the median and the lower and upper borders represent the 1st and 3rd quartiles. The upper whisker shows either the 3rd quartile plus 1.5 times the interquartile range or the maximum score. The lower whisker represents either the 1st quartile minus 1.5 times the interquartile range or the minimum score. Median values and interquartile range of the study cohort: total IQ = 98.0, 85.8–107.0, verbal comprehension = 96.5, 88.0–103.5, perceptual reasoning = 102.0, 94.0–110.5, working memory = 96.0, 82.0–102.0 and processing speed = 94.0, 88.0–103.0. Lines in the background represent normative values provided by the manual (*M*: 100, SD ± 15). Significant differences of the mean of total IQ and the 4 indices compared to the test norm values are marked as follows: *P < 0.05, **P < 0.01, ***P < 0.001. For *M* and SD of the study cohort, please refer to the text.

Results for IQ are shown in Fig. 2. Whereas verbal comprehension (96.8 ± 12.5, *P* = 0.061) and perceptual reasoning (100.9 ± 14.8, *P* = 0.610) did not differ from the normative sample (100 ± 15), total IQ (96.0 ± 13.5, *P* = 0.023), working memory (93.6 ± 13.7, *P* < 0.001), and processing speed (95.4 ± 13.0, *P* = 0.008) were significantly lower. The prevalence of children with an IQ score ≤ 85 was not significantly different from the normative sample (15.9%) for perceptual reasoning (14.9%, *P* = 0.765), processing speed (17.9%, *P* = 0.518), or verbal comprehension (15.7%, *P* = 0.950). A significant difference to the normative sample was found for total IQ (24.6%, *P* = 0.006) and working memory (32.8%, *P* < 0.001).

**Fig. 3:**
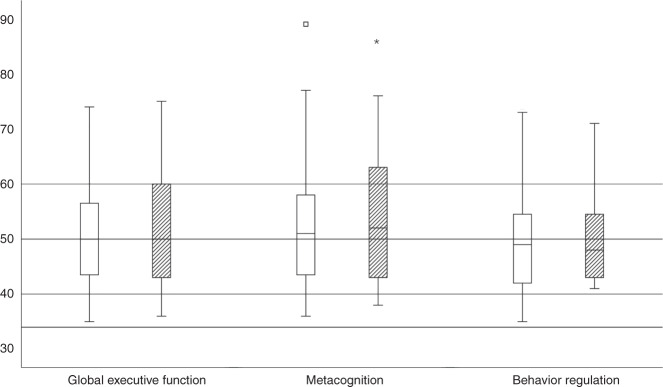
Executive functions measured with parent- and teacher-reported BRIEF. Boxplots represent executive functions of the study population measured with the BRIEF. Parent-reported EF as white boxplots and the teacher-reported ones are patterned. The line within the box is the median and the lower and upper borders represent the 1st and 3rd quartiles. The upper whisker shows either the 3rd quartile plus 1.5 times the interquartile range or the maximum score. The lower whisker represents either the 1st quartile minus 1.5 times the interquartile range or the minimum score. Median values and interquartile range of the study cohort measured with parent-reported BRIEF: global executive functions = 50.0, 43.8–56.5, metacognition = 51.0, 43.0–58.0, and behavioral regulation = 49.0, 42.0–55.0. Median values and interquartile range of the study cohort measured with teacher-reported BRIEF: global executive functions = 50.0, 43.0–60.0, metacognition = 52.0, 43.0–63.0, and behavioral regulation = 47.5, 43.0–55.3. The lines in the background represent normative values provided by the manual (M: 50, SD ± 10). Significant differences of the means of executive functions compared to the test norm values are marked as follows: *P < 0.05, **P < 0.01, ***P < 0.001. For M and SD of the study cohort, please refer to the text.

Results for executive functions assessed with the BRIEF are shown in Fig. 3. Parent-reported executive functions did not significantly differ from the normative values (50 ± 10) for global executive functions (51.3 ± 9.9, *P* = 0.332), behavioral regulation (49.8 ± 9.5, *P* = 0.882), or metacognition (52.5 ± 11.3, *P* = 0.077). Teacher-reported metacognition scores (53.2 ± 10.5, *P* = 0.023) were higher (indicating poorer executive functions) in children with CHD compared to the normative values, while teacher-reported global executive functions (52.3 ± 10.3, *P* = 0.095) and behavioral regulation (50.5 ± 9.3, *P* = 0.678) were similar. Increased proportions of executive dysfunctions, defined as *T* ≥ 65 (norm 6.7%), were found for the following indices: parent-reported global executive function (12.2%, *P* = 0.019), metacognition (16.5%, *P* < 0.001), teacher-reported global executive function (15.5%, *P* < 0.001), metacognition (20.7%, *P* < 0.001) and behavioral regulation (11.8%, *P* = 0.032).

When examining the role of medical risk factors and SES on IQ, the regression model as a whole was significant (*R*^2^ = 0.293, *P* < 0.001). IQ at 10 years of age was significantly predicted by SES (*β* = 0.346, CI-95 = 1.197 to 3.299, *P* < 0.001) and length of hospitalization after the first surgery (*β* = −0.252, CI-95 = −0.190 to −0.026, *P* = 0.011) but by none of the other risk factors (Supplementary Table S1). When examining the role of medical risk factors and SES on parent- and teacher-reported executive functions, no regression model was significant as a whole (all *P* > 0.205).

#### Type and level of education and educational support

Overall, 82.4% of the children with CHD attended regular school. In comparison, the Federal Statistical Office of Switzerland reports that 97% of children aged 6–12 years attend regular school (*P* < 0.001). Of those children with CHD who attended regular school, 26.4% needed additional educational support (i.e., additional kindergarten year: 15.5%, grade retention: 4.9%, introductory/transition class: 11.7%, or additional tutoring: 14.6%). Additionally, there were significantly more CHD children with special education needs integrated into regular education classrooms (8.8% versus 0.7%, *P* < 0.001) or school for children with special needs (8.8% versus 1.7%, *P* < 0.001) than the representative Swiss children.

#### Participation in leisure activities

Ninety children (72.0%) were involved in one or more sport activity at least every other week. In comparison, the Sport Switzerland study reports that 74.0% of all Swiss children aged 10–14 years participate in sport activities at least 3 h per week. Sports activities in the CHD cohort included gymnastics (29.9%), soccer (26.6%), horse-riding (14.4%), swimming (11.0%), dancing (8.9%), floorball (7.8%) or other sports (22%; e.g., martial arts, tennis, or golf). Further, 46.0% of the CHD children were involved in musical activities (e.g., played a musical instrument, sang in a choir) and 20.8% participated in other social activities, for example, were involved in scouts. No representative Swiss data was available with which these results could be compared. Summing participation across all activities showed that CHD children’s participation in leisure activities ranged from zero to five activities, with a median of one activity.

### Impact of neurodevelopmental problems on educational outcome and participation in leisure activities

Type and level of education was predicted by total IQ but not parent-reported global executive functions after correcting for SES (IQ: *B* = 0.135, CI-95 = 0.091 to 0.180, *P* < 0.001, global executive functions *B* = −0.010, CI-95 = −0.056 to 0.035, *P* = 0.648). In a model with teacher-reported global executive functions, both total IQ and executive functions predicted the overall type and level of education after correcting for SES (IQ: *B* = 0.152, CI-95 = 0.098 *to* 0.206, *P* < 0.001, global executive functions *B* = −0.079, CI-95 = −0.132 *–* −0.026, *P* = 0.004).

Participation in leisure activities was predicted by total IQ but neither parent- nor teacher-reported global executive functions after correcting for SES (model with parent-rated executive functions: IQ: *B* = 0.036, CI-95 = 0.005 to 0.067, *P* = 0.022), global executive functions *B* = −0.028, CI-95 = −0.068 to 0.012, *P* = 0.173; model with teacher-rated executive functions: (IQ: *B* = 0.055, CI-95 = 0.019 to 0.091, *P* = 0.003, global executive functions *B* = −0.021, CI-95 = −0.062 to 0.021, *P* = 0.337).

## Discussion

This study reports IQ and executive function profiles, type and level of education and educational support, and participation in leisure activities in a contemporary cohort of 10-year old children with CHD who underwent CPB in the first 6 years of life. Total IQ, working memory, and processing speed measured with the WISC-IV,^22^ and metacognitive abilities measured with the teacher-reported BRIEF^23^ were all lower than the respective norms. Fewer children with CHD attended regular school than the general Swiss population and many required additional educational support, including additional years of schooling. Children with CHD in this cohort actively participated in leisure activities by taking part in sports and music or other activities. Lower IQ and poorer executive functions were associated with more educational support and lower IQ was associated with less participation in leisure activities.

Some studies have shown mean IQs in the low-to-average range in children with CHD at school age with 5–10 IQ points lower than the norm population,^2–7^ whereas others have reported no IQ differences between CHD patients and norm values or compared to typically-developing children.^32,33^ In our cohort, total IQ was significantly lower than the norm, with a mean difference of 4–7 IQ points. Working memory and processing speed were particularly affected. Importantly, at 6 years of age, neither processing speed nor working memory had been different from the norm in this cohort.^2^ This suggests that some neurodevelopmental problems may only become apparent as children with CHD become older. Our findings, thus, underline the necessity for future studies to assess individual longitudinal trajectories of cognitive development in patients with CHD into later school age and adulthood.

Interestingly, the domains found to be most affected in our cohort of children with CHD, namely, processing speed and working memory, are considered cognitive proficiencies.^34^ They are thought to be essential for efficient information processing, particularly in higher-order cognitive tasks such as problem-solving and reasoning, and thus, may be considered core elements of executive functioning.^35^ In contrast, the domains which were comparable to the norm in our cohort of children with CHD, namely, verbal comprehension and perceptual reasoning, are considered general intellectual abilities and estimate IQ with less reliance on cognitive proficiencies.^34^ These children may, thus, have the abilities necessary to learn; however, they may need additional educational support and possibly more time to translate their abilities into educational success. The increased amount of educational support and additional years of schooling described for our cohort reinforce this interpretation.

Alongside IQ, the current study assessed executive functions because they have been shown to play an important role in everyday life, educational outcome, health, prosperity, and quality of life.^36,37^ In the current study, teacher-reported metacognition was significantly worse in children with CHD compared to the norm whereas no difference was found for the global executive functions or behavioral regulation index. Importantly, however, in all but the parent-reported behavioral regulation index, significantly more children were at or above the clinical cut-off for impairments compared to the norm. These findings are in line with previous studies reporting weaknesses in a variety of executive functions in children with CHD.^5,9,10^

When examining the educational situation of our cohort, we found that 85.4% of the children with CHD were enrolled in regular schools. This is considerably less compared to the 97% of children in the general population of Switzerland. This finding is in line with findings from Germany (83.4%)^38^ and another study from Switzerland reporting on a cohort born approximately ten years prior to the current cohort (88%).^39^ Importantly, we found that even those children who attended regular school were frequently in need of additional educational support, including grade retention or an additional year of kindergarten. Along the same line, Riehle-Colarusso and colleagues found that also children with mild forms of CHD were in need of more educational support than healthy controls.^40^

Encouragingly, children with CHD in our study cohort actively participated in leisure activities: The majority of all children with CHD were engaged in some kind of sports, were involved in social activities such as scouts, or played a musical instrument. While no norms are available for social and musical activities, participation in sports is comparable to Swiss children in general.^28,29^ Previously, adolescents with CHD have been reported to have limited engagement in active-physical leisure activities; they were most engaged in social (e.g., hanging out with friends) and recreational (e.g., arts) activities.^20^ In line with what we report for 10-year-old children with CHD, another study reported that 77% of adolescents and young adults with CHD participate in competitive or recreational sports, with increased participation being associated with better quality of life.^41^ More studies are needed to investigate participation in leisure activities beyond sports and how participation may change as children with CHD reach adolescence and adulthood.

As shown previously in typically-developing children,^13–16^ we found IQ and executive functions to impact everyday life outcome in children with CHD: First, total IQ and teacher-reported global executive functions predicted the type and level of education and educational support after adjusting for SES. These results are in line with a previous study that showed that not only IQ but also executive functions were important for school competency in children with CHD.^5^ While in that study, parent-reported global executive functions were predictive, in the current study, only teacher-reported executive functions were. Similarly, a study in preterm children found that teacher-reported but not parent-reported global executive functions predicted learning outcomes.^42^ This may reflect the fact that teachers are better able to report on executive functions as they observe the children in situations where executive function deficit may become particularly apparent. More specifically, Cassidy and colleagues suggest that metacognitive difficulties are more apparent in the school environment and therefore reported as worse by teachers than by parents, which is in line with our findings.^10^ Secondly, we found that IQ, but not executive functions were associated with participation in leisure activities in children with CHD. A previous study reported similar findings for adolescents with CHD with ongoing cognitive and motor developmental impairments to be associated with decreased active-physical and social activity participation.^20^ The finding of the current study implicates that patients with CHD should receive continuous, long-term comprehensive follow-up assessments. With that, support for the child and the families may be provided to ensure optimal participation in everyday life. A recommendation for such a framework has recently been published by Ilardi and colleagues.^43^

Importantly, we confirmed the findings of previous studies^21,44–46^ that SES is an important risk factor for lower IQ in children with CHD. In contrast to a previous study,^39^ the complexity of heart disease was not found to be predictive of either IQ or executive functions in the current study. However, longer length of hospitalization after the first surgery predicted lower IQ at 10 years of age. This suggests that postoperative complications rather than the complexity of the heart defect itself may contribute to lower IQ.

### Limitations

The present study has some limitations: The 10-year assessment only included children without genetic or dysmorphic disorders, which explains the relatively good outcome compared to the population of patients with CHD as a whole. These exclusion criteria enabled the evaluation of the impact of the CHD itself on the neurodevelopmental outcome rather than the confounding effects of genetic or dysmorphic disorders. Also, those children who participated in the 10-year follow-up assessment had higher IQ at 6 years of age compared to those children who did not participate. Thus, the current study cohort constitutes a relatively high-functioning group of children with CHD. Another limitation concerns the measurement of executive functions with only questionnaires and without neuropsychological assessments. Similarly, in the current study, only the type and level of education and additional educational support were assessed while no measures of academic performance were available. Future studies should further investigate this to provide a more detailed view on academic difficulties in children with CHD. Lastly, we did not include a control group of healthy, typically-developing children or peers. Instead, the results of children with CHD were compared against normative values for the IQ and executive functions and to population data provided by the Federal Statistical Office of Switzerland for type and level of education and the Sport Switzerland study for participation in sports. A control group would have allowed for a comparison with a better matching group of children, particularly regarding participation in leisure activities.

## Conclusion

To conclude, we demonstrate in a contemporary cohort of children with CHD examined at 10 years of age that total IQ, working memory, processing speed, and metacognition are significantly poorer than the normative values. These deficits affect educational performance and participation in leisure activities. The findings of the current study highlight the importance of a continuous monitoring as children develop and the need to investigate cognitive interventions that effectively reduce the obstacles they face in everyday life.

### Supplementary information

Supplementary information
