# Supplementary material for: Congenital heart disease in school-aged children: Cognition, education, and participation in leisure activities
Source: Pediatr Res. 2021 Dec 1;94(4):1523–9. doi: 10.1038/s41390-021-01853-4 (PMC10589091; doi:10.1038/s41390-021-01853-4)
Supplement: Supplementary file 1 — Supplementary information [file 41390_2021_1853_MOESM1_ESM.docx]

**Supplemental Table S1: Risk factor analysis for IQ of 10-year old children with CHD**

| ***Dependent variable*** | ***Independent variable*** | ***β*** | ***CI-95*** | ***p-value*** |
| --- | --- | --- | --- | --- |
| **IQ** | Socioeconomic status | .346 | 1.20 to 3.30 | <.001 |
| **(total score)** | gestational age (GA, in weeks) | .147 | -.11 to 2.08 | .078 |
|  | birth weight (z-score corrected for GA) | .055 | -1.31 to 2.62 | .513 |
|  | univentricular CHD (yes/no) | -.037 | -6.98 to 4.56 | .679 |
|  | mean preoperative saturation (in %) | .117 | -.08 to .39 | .182 |
|  | age at first CPB (in months) | -.089 | -.87 to .27 | .303 |
|  | lowest perioperative temperature (in °C) | -.091 | -.90 to .31 | .335 |
|  | extracorporeal circulation during the first CPB surgery (in minutes) | -.135 | -.06 to .01 | .158 |
|  | length of hospitalization (in days) | -.252 | -.19 to -.03 | .011 |
